# Squamous cell carcinoma of the nipple following radiation therapy for ductal carcinoma *in situ*: a case report

**DOI:** 10.1186/1752-1947-4-186

**Published:** 2010-06-21

**Authors:** Catherine E Loveland-Jones, Fengwei Wang, Robin R Bankhead, Yajue Huang, Kathleen J Reilly

**Affiliations:** 1 Department of Surgery, Temple University Hospital, 3401 North Broad Street, Philadelphia, PA, 19140, USA; 2 Department of Pathology, Temple University Hospital, 3401 North Broad Street, Philadelphia, PA, 19140, USA

## Abstract

**Introduction:**

Radiation-induced nonmelanoma skin cancer was first reported seven years after the discovery of X-rays, but has received relatively little consideration in the literature. Specifically, nonmelanoma skin cancer after conservative surgery and radiation for early stage breast cancer has not been well studied. We report the case of a woman who developed squamous cell carcinoma of the nipple nine years after conservative surgery and radiation for ductal carcinoma *in situ *of the ipsilateral breast. We also review the relevant literature available to date.

**Case presentation:**

A 66-year-old African-American woman presented to the hospital with a non-healing ulcer of the right nipple. Her past medical history was significant for right breast ductal carcinoma *in situ *for which she had undergone lumpectomy and whole breast radiation therapy nine years previously. Mammography and magnetic resonance imaging studies were negative for recurrent breast cancer. However, the latter demonstrated abnormal enhancement in the nipple-areolar region. An incisional biopsy of the lesion demonstrated invasive squamous cell carcinoma. Subsequently, the patient underwent wide excision of the nipple-areolar complex. Sentinel lymph-node biopsy was offered but our patient declined. She was considered to have local disease and hence no further treatment was recommended.

**Conclusion:**

This case represents the first reported occurrence of squamous cell carcinoma of the nipple to follow conservative surgery and radiation for ductal carcinoma *in situ *of the ipsilateral breast. It is likely that radiation overexposure resulted in a radiation burn and subsequent radiodermatitis, placing it at risk for squamous cell carcinoma. A diagnosis of squamous cell carcinoma should be considered in a patient with a nipple lesion following radiation therapy for breast cancer.

## Introduction

Breast-conserving surgery followed by whole breast radiation is a common mode of treatment for breast cancer, and is equivalent to mastectomy in the treatment of early stage disease [1,2]. Radiation has no detrimental effects on breast cancer survival and, as recently reported, may actually increase survival when given optimally in accordance with modern dose and target recommendations [3]. Nevertheless, radiation has been reported in several studies to increase the risk of second non-breast malignancies, including leukemia, sarcoma, lung cancer, and esophageal cancer. In one report comparing breast cancer patients who received radiation therapy after surgery with those who did not, the relative risks of lung cancer and myeloid leukemia were 1.62 and 2.99, respectively [4]. The development of these second malignancies is influenced by patient factors such as age, tobacco use, and history of adjuvant systemic therapy [5,6].

Radiation-induced nonmelanoma skin cancer (NMSC) was first reported seven years after the discovery of X-rays [7]. Despite this long established relationship, NMSC is not consistently included in studies evaluating second non-breast malignancies that follow conservative surgery and radiation for breast cancer. We present the case of a woman who developed squamous cell carcinoma (SCC) of the nipple nine years after conservative surgery and radiation for ductal carcinoma *in situ *(DCIS) of the ipsilateral breast.

## Case presentation

**Figure 1:**
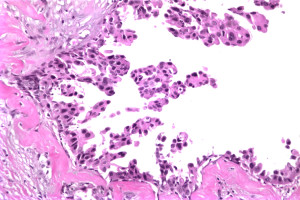
**Right breast DCIS, micropapillary type**.

In December 2007, a 66-year-old African-American woman with a past medical history significant for diabetes mellitus, hypertension, congestive heart failure, and fibroids presented to the Temple University Hospital, Philadelphia with a non-healing ulcer of the right nipple. The patient’s past medical history was also significant for right breast DCIS for which she had undergone lumpectomy and whole breast radiation therapy in February 1998. These interventions were followed by five years of tamoxifen therapy. By mammography, the DCIS lesion measured three centimeters and was located six centimeters from the nipple in the inferomedial quadrant of the right breast. At lumpectomy, the lesion was a nuclear grade II/III micropapillary tumor with apocrine features (Figure 1). The margins of the specimen were negative for tumor.

Our patient received a standard course of radiation. Over 34 days, the right breast was treated with 6 Mv photons to a dose of 5000 cGy using medial and lateral tangential fields measuring 16 × 22 cm. Fifteen degree wedges were used to enhance dosimetry. The daily fraction size was 200 cGy. Over four days, the area of resection was boosted an additional 1000 cGy using an en face 16 MeV electron field measuring 12 × 5 cm. The total dose of radiation was 6000 cGy.

**Figure 2:**
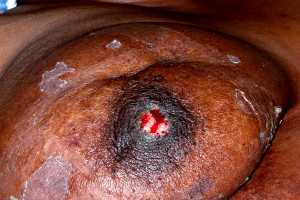
**Gross depiction of nipple ulcer**.

Nine years after surgery and radiation, our patient presented with a non-healing ulcer of the right nipple (Figure 2). The lesion was an irregularly shaped excoriation that partially eroded through the nipple. There was both serous and bloody discharge. The surrounding nipple-areolar tissue was effaced, retracted, and indurated. There was no odor or cellulitis. She stated she had been suffering from a "raw" right nipple with intermittent healing for approximately two years.

There were no other significant physical exam findings, including no palpable axillary lymphadenopathy. Mammography and magnetic resonance imaging studies were negative for recurrent breast cancer. However, the latter demonstrated abnormal enhancement in the nipple-areolar region.

**Figure 3:**
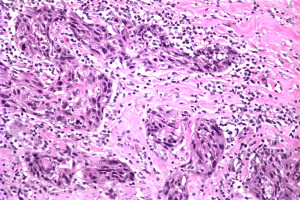
**Right breast moderately to poorly differentiated invasive SCC**.

**Figure 4:**
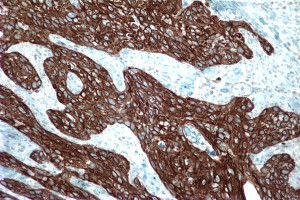
**CK903 immunostaining of SCC**.

**Figure 5:**
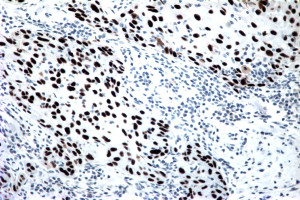
**p63 immunostaining of SCC**.

An incisional biopsy of the lesion demonstrated invasive SCC. Subsequently, our patient underwent wide excision of the nipple-areolar complex. Pathologically, the lesion measured 15 mm (length) × 14 mm (width) × 9 mm (depth) and was characterized as a moderately to poorly differentiated invasive SCC (Figure 3). Immunohistochemical staining was positive for CK903 (Figure 4), CK5/6, p63 (Figure 5), and calponin and negative for estrogen and progesterone receptors as well as Her-2/neu, supporting a diagnosis of SCC. The resection margins were free of tumor and there was no evidence of lymphovascular invasion. The tumor was staged T1N0M0 (Stage 1).

Our patient was felt to be at high risk for recurrence according to the National Comprehensive Cancer Network Clinical Practice Guidelines [8]. Specifically, her tumor occurred at the site of prior radiation therapy and had poorly defined borders, a depth greater than 4 mm, and a moderate to poor differentiation. For this reason, re-excision of the area was performed to ensure minimum 10 mm margins were obtained. Sentinel lymph-node biopsy was offered but our patient declined.

She was considered to have local disease and hence no further treatment was recommended. She will have close clinical follow-up biannually and mammography annually. She was also instructed to perform self skin and lymph node exams.

## Discussion

Multiple risk factors for SCC have been defined. The most significant of these is solar radiation exposure. Multiple studies have shown the incidence of SCC increases with proximity to the equator and cumulative sun exposure [9]. Others risk factors include fair coloring, genetic disorders of photosensitivity (e.g. xeroderma pigmentosum), chronic skin conditions (e.g. burn scars and venous ulcers), impaired immunity, and exposures (e.g. arsenic and radiation therapy).

Radiation therapy has also been described as a risk factor for SCC. In a report from the Childhood Cancer Survivor study, radiation therapy was associated with a 6.3 fold-increase in risk for NMSC (SCC and basal cell carcinoma). Of these cancers 90% occurred within the radiation field [10]. The association between radiation and NMSC is also prevalent in the head and neck literature.

SCC subsequent to radiation therapy appears to be more aggressive than that due to sunlight. In one study, SCC arising in previously burned or irradiated skin was associated with a five-year survival of only 52%. This was in contrast to the 90% five-year survival observed in patients with sunlight-induced SCC. Proposed explanations of this difference included greater biologic aggressiveness of scar SCC, delayed presentation, and areas of scarring being immunologically privileged sites [11].

Despite an extensive literature search, no reports of SCC of the nipple following radiation therapy for breast carcinoma were found. This result is surprising given the large number of patients undergoing radiation therapy for breast cancer. Overall, SCC of the nipple is quite rare, likely because the area is not a sun-exposed region of the body.

Multiple technical factors during radiation therapy for breast cancer influence the risk of second malignancies. These include quality, total dose, fractionation, degree of overlap between the fields, tissue volume in the radiation field, as well as the degree and quality of scatter outside the radiation field [6,12]. The nipple is at significant risk for exposure to toxic doses of radiation if it protrudes into the radiation field unshielded by other tissue. In effect, it may become a "hotspot". Furthermore, inconsistent positioning of the nipple places it at further risk for radiation overexposure. Our patient has large, pendulous breasts, making inconsistent positioning more likely. Exposure of the normal nipple tissue to high doses of radiation may result in radiodermatitis, a risk factor for SCC. Fortunately, improvements in radiation technique over the last decade have decreased the risk of overexposure to the nipple and acute radiation skin toxicity [13].

It is important to keep in mind that the absolute risk of death from a second malignancy following radiation therapy for breast cancer is less than 1% per year [6]. This risk is much smaller than the survival benefit derived from radiation therapy, which is about 6% at ten years [3].

SCC of the nipple should be included in the differential diagnosis of a nipple lesion in a patient presenting after radiation therapy. Other diagnoses to consider include basal cell carcinoma, recurrent breast cancer, Paget's disease, and radiodermatitis.

Treatment options for SCC depend on the risk of recurrence and the presence of positive lymph nodes. Risk factors for recurrence include large diameter (≥20 mm on the trunk and extremities, ≥10 mm on the cheeks, forehead, scalp, and neck, or ≥6 mm on the face, genitalia, hands, and feet) poorly-defined borders, recurrence, immunosuppression, occurrence at the site of prior radiation therapy or chronic inflammation, rapid growth, neurologic symptoms, moderate or poor differentiation, adenoid/adenosquamous/desmoplastic subtypes, greater depth (Clark levels level IV/V or ≥4 mm), and perineural or vascular involvement [8].

Patients without any of these risk factors are at low risk for recurrence and may be treated with field therapies such as curettage and electrodessication. Otherwise, both low and high risk patients are treated surgically with assessment of margins. Surgical options include standard excision with post-operative margin assessment or Mohs micrographic excision using frozen section. Margin adequacy depends on recurrence risk status, size, and location but generally falls between 4 mm and 10 mm. Patients with positive lymph nodes, positive margins after surgery, extensive nerve involvement, and those who are not surgical candidates should be treated with radiation therapy [8].

## Conclusions

This case represents the first reported occurrence of SCC of the nipple to follow conservative surgery and radiation for DCIS of the ipsilateral breast. It is likely that radiation overexposure resulted in a radiation burn and subsequent radiodermatitis, placing it at risk for SCC. A diagnosis of SCC should be considered in a patient with a nipple lesion following radiation therapy for breast cancer.

## Abbreviations

DCIS: ductal carcinoma *in situ*; NMSC: nonmelanoma skin cancer; SCC: squamous cell carcinoma.

## Competing interests

The authors declare that they have no competing interests.

## Authors' contributions

CLJ reviewed the literature and wrote the manuscript. KJR carried out the operation on the patient and was the primary editor of the manuscript. RRB assisted in the care of the patient and edited the manuscript. FW and YH provided the pathology images and interpretation. All authors read and approved the final manuscript.

## Consent

Written informed consent was obtained from the patient for publication of this case report and any accompanying images. A copy of the written consent is available for review by the journal's Editor in Chief.
